# Native aortic valve pneumococcal endocarditis - fulminant presentation

**DOI:** 10.5935/0103-507X.20160004

**Published:** 2016

**Authors:** Kevin Domingues, Liliana Marta, Isabel Monteiro, Margarida Leal

**Affiliations:** 1 Hospital Distrital de Santarém - Santarém, Portugal.

**Keywords:** Endocarditis, bacterial/diagnosis, Pneumococcal infections/diagnosis, Aortic valve, Case reports

## Abstract

Pneumococcal endocarditis is a rare entity, corresponding to 1 to 3% of native valve endocarditis cases. It has a typically adverse prognosis, with high mortality. There is a reported predilection for the aortic valve; thus, a common presentation is acute left heart failure. We present a case of a 60-year-old woman with a history of sinusitis, who was admitted with the diagnosis of pneumonia. She rapidly deteriorated with signs of septic shock and was transferred to the critical care unit. The transesophageal echocardiogram revealed severe aortic regurgitation due to valve vegetations. Blood cultures were positive for *Streptococcus pneumoniae*. She underwent cardiac surgery and had multiple postoperative complications. Nonetheless, the patient made a slow and complete recovery. Infectious endocarditis should be ruled out if any suspicion arises, and echocardiography should be performed in an early stage in patients with poor response to vasopressors and inotropes. Patients with pneumococcal endocarditis benefit from an aggressive approach, with performance of early surgery.

## Abstract (pt)

A endocardite pneumocócica é uma entidade rara, cuja incidência se situa entre 1% e 3% dos casos de endocardite de válvula nativa. Esta patologia tem um prognóstico naturalmente adverso, com elevada mortalidade. Relata-se predileção pela válvula aórtica, de forma que é frequente que se apresente com insuficiência cardíaca. Apresentamos o caso de uma paciente do sexo feminino com 60 anos de idade e história pregressa de sinusite, admitida com diagnóstico de pneumonia. Após rápida deterioração, com sinais de choque séptico, ela foi transferida para a unidade de terapia intensiva. O ecocardiograma transesofágico revelou grave refluxo aórtico, devido à presença de vegetações valvares. As hemoculturas foram positivas para *Streptococcus pneumoniae*. A paciente foi submetida à cirurgia cardíaca e apresentou múltiplas complicações pós-operatórias. Apesar disso, apresentou lenta, porém completa recuperação. A endocardite infecciosa deve ser afastada em caso do surgimento de qualquer suspeita, e a ecocardiografia deve ser realizada precocemente nos pacientes com resposta insuficiente aos vasopressores e inotrópicos. Pacientes com endocardite pneumocócica se beneficiam de uma abordagem agressiva, com realização precoce da intervenção cirúrgica.

## INTRODUCTION

Pneumococcal endocarditis is a rare type of endocarditis, corresponding to only 1 to 3% of native valve endocarditis cases.^(1,2)^ It has a typically adverse prognosis, with a high mortality rate. The aortic valve is the most commonly affected site. We present a patient who was initially admitted to our hospital with the diagnosis of community-acquired pneumonia, but whose rapid deterioration prompted us to perform echocardiography, revealing the true diagnosis.

## CASE REPORT

**Figure 1:**
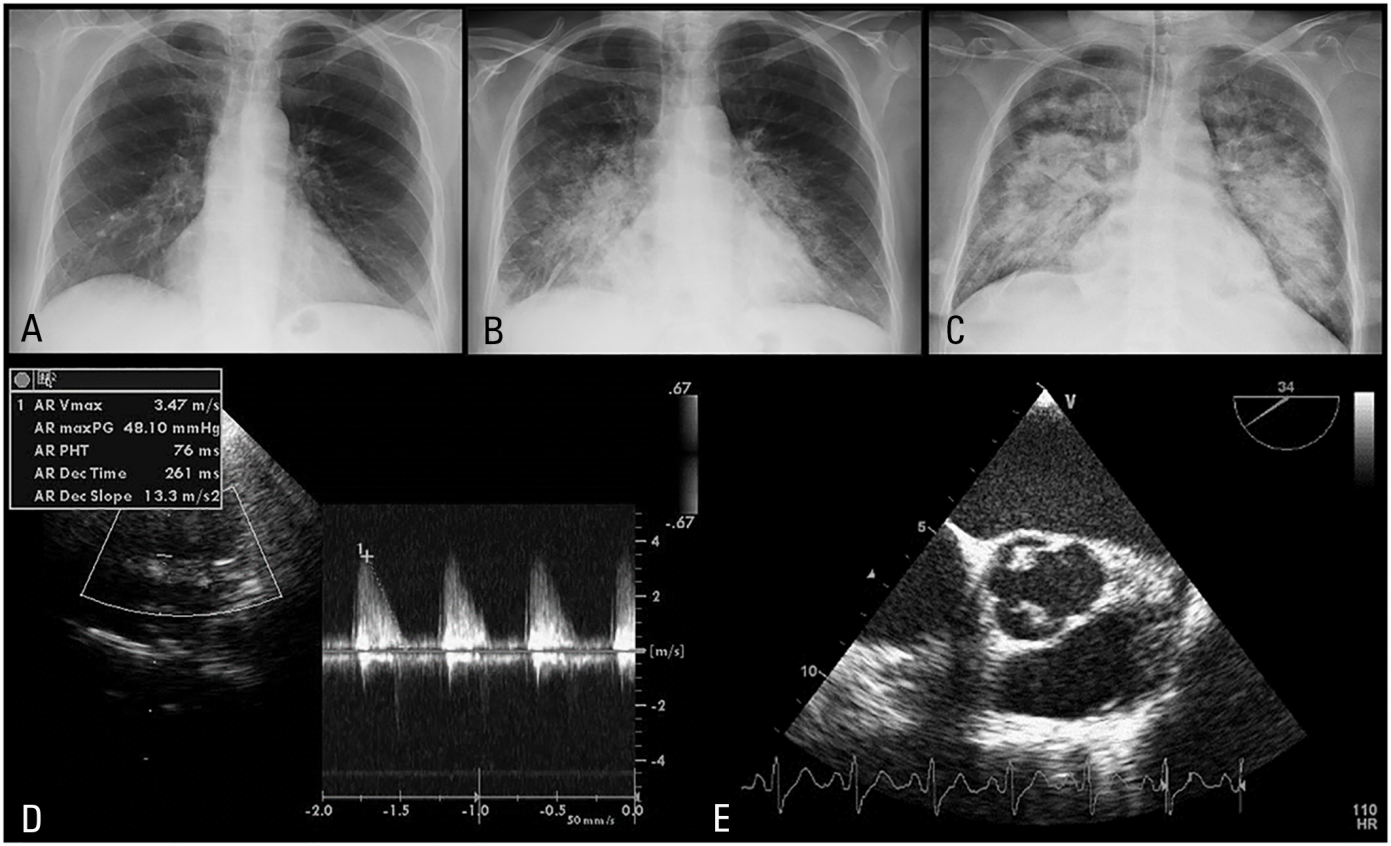
(A to C) - Radiologic evolution - progressive worsening of pulmonary interstitial edema. (D) - Transthoracic echocardiography - severe aortic regurgitation measured by continuous Doppler. (E) - Transesophageal echocardiography - aortic valve vegetations on the non-coronary and right coronary cusps.

A 60-year-old woman with a history of chronic sinusitis presented to the emergency department with a twelve-day history of postprandial vomiting and dyspnea, dry cough, epigastric pain, and malaise for 1 day. She was febrile (38.5ºC), tachycardic (110bpm - sinus rhythm on the electrocardiogram), but her blood pressure was normal. Thoracic auscultation revealed right lung base crackles and no heart murmurs. She had high inflammatory parameters and mild hypoxemia, and the admission chest radiography showed right hilar congestion (Figure 1A). She was admitted to the Internal Medicine ward with the diagnosis of community-acquired pneumonia. Blood cultures were sampled, and she was empirically medicated with ceftriaxone and azithromycin. Over the next 2 days, she deteriorated rapidly, with progressive worsening of clinical, analytical (Table 1), and radiological (Figures 1A to 1C) parameters. She fulfilled the criteria for septic shock and acute respiratory distress syndrome, and she was transferred to the critical care unit, where mechanical ventilation and vasopressor/inotropic support were started. Because there was no improvement, transthoracic echocardiogram was performed the following day and despite bad image quality, showed an important aortic regurgitation (Figure 1D). The transesophageal echocardiogram confirmed severe aortic regurgitation and the presence of two aortic valve vegetations, adherent to the right coronary and to the non-coronary cusps (Figure 1E). Empirical therapy with weight-adjusted vancomycin and gentamycin was then started. Blood cultures were positive for *Streptococcus pneumoniae*, which is sensitive to both antibiotics; bronchial aspirate cultures were negative. Despite 6 days of directed therapy, the patient remained febrile and dependent of inotropic support, and was therefore transferred to a cardiac surgery center, where massive valve destruction was confirmed. The aortic valve was resected and replaced with a tissue prosthetic valve, and the antibiotic was changed to intravenous penicillin. The postoperative period was complicated by cardiogenic shock (with an initial cardiac index of 0.9L/min/m^2^ and an estimated left ventricular ejection fraction of 20% due to diffuse hypokinesis, with a good response to inotropic support), atrial fibrillation with rapid ventricular response, acute kidney injury (with the need of continuous venovenous hemodiafiltration), and ventilator-associated pneumonia (with *Morganella morganii* as the causative organism). Although the patient's recovery was slow, it was complete, and she was discharged forty days after her initial admission. Her clinical and functional status was excellent at the 3 month follow-up. Her most recent echocardiogram showed normal left ventricular systolic function and a normally functioning prosthetic aortic valve. Pneumococcal vaccination was later administered.

**Table 1:** Evolution of analytical parameters during hospitalization

	Day 1	Day 2	Day 2	Day 3	Day 4	Day 5	Day 6	Day 7	Day 8
Event	Admission		Transfer to ICU	D0 vancomycin and gentamicin					Transfer to cardiac surgery unit
Hemoglobin (g/dL)	11.5	10.5	9.5	9.9	9.4	7.9	9.6	8.9	9.3
WBC (x10^9^/L)	13.3	17.4	17.2	26.5	18.3	16.6	16.2	15.3	13.3
Platelets (x10^9^/L)	167	147	145	215	215	252	332	258	301
Creatinine (mg/dL)	0.9	0.8	0.8	0.7	0.7	0.9	0.9	1.0	1.1
CRP (mg/dL)	21.0	27.6	28.8	24.1	21.1	16.5	12.1	7.2	6.3
Lactate	1.3	3.4	4.7	2.1	1.7	1.4	1.8	2.5	1.8
pH	7.43	7.45	7.36	7.38	7.44	7.38	7.44	7.51	7.43
PaCO_2_ (mmHg)	32.7	26.1	34.4	34.6	32.2	42.5	36.1	33	43.1
PaO_2_ (mmHg)	60.2	49.1	64.6	81	92.1	79.6	83.2	72.2	84.5
PaO_2_/FiO_2_	193	82	81	101	115	114	119	120	141
SBE	-2.6	-5.4	-5.8	-3.8	-1.4	-0.4	0.4	4	4.1

DO - day zero; WBC - white blood cell count; CRP - C-reactive protein; PaCO_2_ - arterial carbon dioxide partial pressure; PaO_2_ - arterial oxygen partial pressure; PaO_2_/FiO_2_ - ratio of arterial partial pressure to inspired oxygen fraction; SBE - standard base excess.

## DISCUSSION

*Streptococcus pneumoniae* is an unusual and rare causative organism of endocarditis in the penicillin era. It has been associated with alcohol abuse, the elderly, and pre-existing valvular heart disease^(1)^ and might present with concomitant pneumonia and meningitis, as part of Austrian syndrome.^(3)^ These conditions did not appear to contribute to pneumococcal endocarditis in this case. Pneumococcal bacteremia is commonly a consequence of lung infection, but rarely, it may follow otitis media and sinusitis (13.7%).^(1)^ Due to its typical acute presentation, peripheral stigmata of infective endocarditis are seldom evident.^(1)^ According to previous reviews, there is a predilection of pneumococcal endocarditis to affect the aortic valve (74.4%); thus, it will commonly present as acute heart failure,^(1,4)^ and be associated with higher mortality.^(5)^ The pursuit of aggressive treatment options is essential in these patients, and a combined medical and surgical approach seems to show improved outcomes.^(5)^

## CONCLUSION

In summary, this case represents the successful treatment of a rare case of aggressive pneumococcal endocarditis, and it demonstrates how it can mimic other diseases, such as pneumonia. High suspicion is essential to facilitate the performance of echocardiography at an early stage and to apply the best treatment strategy. Patients with pneumococcal endocarditis benefit from an aggressive approach, with performance of early surgery.
